# Reappraisal of Frequency of Common Cystic Fibrosis Transmembrane Conductance Regulator Gene Mutations in Iranian Cystic Fibrosis Patients

**Published:** 2018-02

**Authors:** Soheila Khalilzadeh, Maryam Hassanzad, Mihan PourAbdollah Toutkaboni, Sabereh Tashayoie Nejad, Fatemeh-Maryam Sheikholeslami, Ali Akbar Velayati

**Affiliations:** Pediatric Respiratory Diseases Research Center, National Research Institute of Tuberculosis and Lung Disease, (NRITLD), Shahid Beheshti University of Medical Sciences, Tehran, Iran

**Keywords:** Cystic Fibrosis, Gene, Mutation, Iran, Patients, Genotype, Phenotype

## Abstract

**Background:**

Cystic Fibrosis (CF) is a life-threatening recessive genetic disorder resulting from mutations in the gene encoding the fibrosis transmembrane conductance regulator protein (CFTR). The CF clinical phenotype shows wide variation ranging from severe disease in early childhood in those homozygous for the p.Phe508del mutation to absence of the vas deferens in otherwise healthy men homozygous for the p.Arg117His mutation.

**Materials and Methods:**

DNA was extracted from whole blood from 62 patients with CF. The *CFTR* mutation was determined by Allele-Specific PCR assay. The spearman and linear regression analysis were used to obtain the correlation between phenotype and genotype relationship.

**Results:**

Out of total 62 patients, 35 (56.4%) were male. The mean age of the patients was 15.56 ± 6.65 years. Mutations in *CFTR* were detected in 64.5% of the patients. The commonest mutations were p.Phe508del (33.9%), p.Arg117His; [5T] (5.64%), p.Arg117His; [7T] (4.03%) and p.Trp1282X (5.64%). Mutations p.Ile507del (4%), p.Gly542X (4%), p.Asn1303Lys (2.42%), c.489+1G>T (1.6%), p.Gly551Asp (1.6%) and c.1585-1G>A (1.6%) were also detected. Most mutations were detected in west and south of Iran, while p.Phe508del mutation was dominant mutation (75%) in east and southeast of Iran. The study showed either an association between this mutation with severity of disease and sex or an association between p.Arg117His mutations and age at diagnosis.

**Conclusion:**

The geographic distribution of gene mutation in Iranian cystic fibrosis patients was very heterogenic. In spite of the study that showed a correlation between p.Phe508del and severity of disease, to find any correlation between genotype and phenotype a broad and multi-centered study is recommended.

## INTRODUCTION

The Cystic Fibrosis Transmembrane Conductance Regulator (*CFTR*) gene encodes the CFTR protein (1). More than 2000 *CFTR* mutations are associated with abnormal function of the chloride channel (2). Mutation frequencies vary between different ethnic populations based on their geographic distribution (3). Published data suggest that the frequency and distribution of *CFTR* mutations vary in Iranian populations depending on their geographical region and ethnicity (2,4–7).

Generally, the loss of function or expression of *CFTR* causes severe symptoms of Cystic Fibrosis (CF), but mutations that reduce function or expression of *CFTR* cause atypical and mild form of disease (8). However, different studies do not always agree on the relationship between genotype and phenotype in CF (8,9).

A comprehensive study to identify *CFTR* mutations across several ethnic populations is very important and should be considered in health planning. For this reason, this study was conducted to recognize the common 11 *CFTR* gene mutations as well as poly T polymorphisms in 62 CF patients who were registered from the different geographical regions of Iran at the Pediatric Respiratory Disease Research Center which serves as a national referral CF center. Many studies on imaging, clinical, and para-clinical aspects as well as mortality of disease have been done in this center (10–13). Also the correlation of genotype and phenotype of the disease was assessed in these patients.

## MATERIALS AND METHODS

Sixty-two CF patients registered at the Pediatric Respiratory Disease Research Center in Masih Daneshvari Hospital in Tehran were evaluated for the 11 most common *CFTR* mutations as well as poly-T polymorphisms. Patients were enrolled in the study based on elevated sweat chloride levels (>60 meq/l) on two occasions using the pilocarpine iontophoresis method or clinical features and evidence of malabsorption and GI problems. Whilst using Creon 35 patients among 62 (56.45%) suffered from pancreatic insufficiency. Based on the Shwachman-Kulczycki score (14), the patients were categorized as i) Severe: 66.1% (41/62), ii) Moderate: 25.8% (16/62), iii) Mild 6.5% (4/62) and iv) Good: 1.6% (1/62).

### DNA extraction

Genomic DNA was extracted from two ml of whole blood stored in Na-EDTA using a silicon-based extraction kit according to manufacturer instruction (YTZ, IRAN). The concentration and quality of DNA was evaluated by spectrophotometer at 260 and 280 nm, and aliquots stored both at 4°C for immediate use or −20°C for long term storage.

### Mutation analysis

11 common *CFTR* mutations (p.Phe508del, p.Ile507del, p.Arg117His, p.Trp1282X, c.489+1G>T, p.Arg560Thr, p.Gly551Asp, c.1585–1G>A, p.Arg553X, p.Gly542X and p.Asn1303Lys, poly T polymorphisms) were genotyped in this study. Specific oligonucleotide primers sequences, Tm and the band size of each amplicon were described previously (15,16). Amplification was carried out in a volume of 10 μl using genomic DNA (100 ng), forward and allele-specific reverse primers (0.5 pM each), 5 μl of 2X PCR master mix Buffer (YTZ, IRAN), 0.5 μl of 20X EVA green (YTZ, IRAN). PCR was performed in a Corbet thermocycler under following condition: initial denaturation step at 95°C for 15 min, denaturation step at 95°C for 20 sec, annealing step at specific Tm for each set of primers for 15 sec, polymerization step at 72°C for 10 second. The cycles were repeated for 35 times. The acquisition was performed at 72°C. To determine the non-specific band, mismatch and primer dimers, the melting curve was also resolved.

Separate pipettes with disposable barrier tips, and contained physical space were used pre- and post-PCR to prevent the cross contamination between samples. A negative control (distilled water) was included in each step of the procedure. Additionally, all of the tests were performed twice.

### Ethical approval

This study was approved by the Ethics Committee of the National Research Institute of Tuberculosis and Lung Diseases (Iran/Tehran). The approval No.of project was IR.SBMU.NRITLD.REC.1394.158. Informed written consent was obtained from patients or their parents regarding genetic testing for the disease.

### Statistical analysis

The Statistical Package for the Social Sciences v.16.0 (SPSS, Inc., Chicago, IL, USA) software was used for statistical analysis. For descriptive analysis, we used the mean, median and standard deviation for continuous variables and absolute frequency (percentage) for categorical variables. Fisher’s exact test and the χ^2^ test were performed to compare the groups using categorical variables. The correlations (Spearman) and linear regression analysis were used to obtain the correlation between phenotype and genotype relationship.

## RESULTS

### Demographic analysis

In this study 62 CF patients registered at the Pediatric Respiratory Disease Research Center of Iran were evaluated for 11 common *CFTR* mutations and poly-T polymorphisms. The female to male ratio of the patients was 27/35=0.77. The mean and median age of the patients were 15.56 ± 6.65and 16 years, respectively (range: 2 to 33 years). The demographic data of the patients is summarized in table 1.

**Table 1.** Demographic data of CF patients registered in Pediatric Respiratory Research Center of Iran

**Number of patients**			**62 patients (124 alleles)**
**Age**	Mean		6.652±15.56 years
	Median		16 years
	Range		2 to 33 years
**Age group (Years)**	0–14		24 (38.7%)
	15–20		36 (58.1%)
	30 and over		2 (3.2%)
**Sex**	Male		35 (56.45%)
	Female		27 (43.54%)
**Ethnic groups**	Iranian patients		60 (96.77%)
	Non-Iranian patients	Afghan	1 (1.6%)
		Arab	1 (1.6%)
**Age of diagnosis**	Mean		93.415±66.15 months
	Median		10 months
**BMI**	<5th percentile		33 (53.2%)
	Normal		26 (42%)
	Undetermined		3 (5%)
**Sweat test**	>60meq/l		41 (66%)
	=60meq/l		2 (3%)
	undetermined		19 (31%)
**Consanguineous marriage**			46 (74.2%)
**Manifestation**	Bronchiectasis		56 (90.3%)
	Pancreatic insufficiency		35 (56.45%)
	Liver complication		6 (9.67%)
	Nasal polyp		3 (4.8%)
	Diabetes mellitus		8 (13%)
**Meconium ileus**			undetermined
**Bacterial and fungal infectious**	*Pseudomonas aeruginosa*		36 (58.1%)
	*Staphylococcus aureus*		14 (22.6%)
	*Aspergillus spp.*		4 (6.45%)
	*Escherichia coli* & *Enterococcus*		4 (6.45%)
	*Candida spp*		2 (3.22%)

Patients were referred to our center from different regions of Iran. Since Iran has a heterogenic population with different ethnic groups such as Lore, Kurd, Baloch, Azari, Arab and Fars, the patients were categorized into 10 groups based on the geographic regions where they lived and their ethnicity. All the patients had Iranian origin while two patients were categorized as non-Iranian patients who came from Afghanistan and Arabic countries. While Tehran is the capital, its population is heterogenic due to migration from other regions of Iran. Unfortunately, there was no evidence about the origin of the patients who lived in Tehran. Consanguinity was present among 74.2% of parents of the CF patients. All of the families were Muslim.

### Genotype analysis

In total, we found a mutation in at least one allele of *CFTR* in 64.5% of the CF patients, but 22 patients (35.5%) did not carry any of the 11 mutations we tested for. The most frequent mutation detected in the patients was p.Phe508del (42/124 alleles or 33.9%), with 17 (27.4%) patients found to be homozygous and 8 (13%) heterozygous for this mutation. The second most frequent mutation was p.Arg117His (12/124=9.67%) of whom one (1.6%) patient was homozygote. Six of them (54.5%) were p.Arg117His with 5T variant and the others were p.Arg117His with 7T variant. The third most frequent mutation detected in our sample was p.Trp1282X (5.64%). The frequency of p.Ile507del, p.Gly542X and p.Asn1303Lys were more than 2%. The frequencies of mutations in the *CFTR* gene are summarized in table 2.

**Table 2.** Spectrum of CFTR mutations detected in 124 alleles of the CF patients registered at Pediatric Respiratory Disease Research Center of Iran.

	**CDNA name**	**Protein name**	**exon/intron**	**No. of alleles**	**%**
1	C.1521_1523DelCTT	P.Phe508Del	EX10	42	33.9
2	C.1519_1521DelATC	P.Ile507Del	EX10	5	4
2	C.[350G>A;1210–12]	P.Arg117His;[5T]	EX4	7	5.7
3	C.[350G>A;1210–12]	P. Arg117His;[7T]	E4	5	4
4	C.3846G > A	P.Trp1282X	EX20	7	5.7
5	C.1624G > T	P.Gly542X	EX11	5	4.0
6	C.3909C > G	P.Asn1303Lys	EX21	3	2.4
7	C.1652G > A	P.Gly551Asp	EX11	2	1.6
8	C.489 + 1G > T	C621 + 1 G>T	IN4	2	1.6
9	C.1585–1G > A	C1717–1 G>A	IN10	2	1.6
10	C.1657C > T	P.Arg553X	EX11	0	0
11	C.1678A>G	P.Arg560Thr	EX11	0	0
12	Unknown			44	35.5

Twenty-six (41.9%) patients were compound heterozygotes, 8 (30.8 %) of whom had complex genotype.

**Figure 1.**
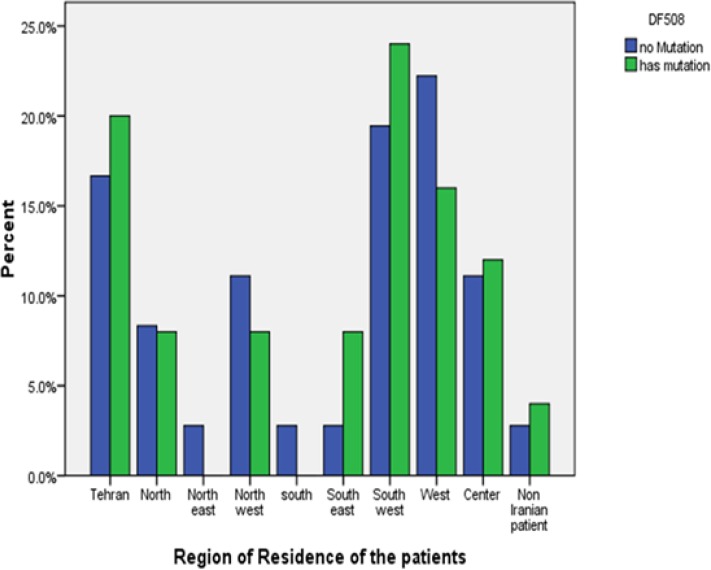
Geographic distribution of P.Phe508del mutation in the CF patients registered at Pediatric Respiratory Disease Research Center of Iran.

The geographic distribution of *CFTR* mutations was evaluated based on the patient’s origins. Most of the mutations were from patients residing in the northwest, west and southwest of Iran. However, the p.Phe508del mutation was commonest in the Baloch ethnic group who lived in the southeast of Iran (66.7% of patients bearing p.Phe508del mutation compared to 40% in patients who lived in the west and southwest regions of Iran, 33.3% in north regions of Iran and 36.8% in the west and central region of Iran(. Even though the p.Phe508del mutation was detected in 45.5% of the CF patients who lived in Tehran, it should be noted that this region of country is heterogenic and different ethnic groups such as Lore, Kurd, Baloch and Turk live in this town (Figure 1).

Chi-square analysis showed that there was a significant difference between the mutation in p.Phe508del with gender of the patients (p<0.032, CI: 95%). Pearson’s Correlation and Regression linear analysis showed that there was a rather strong positive linear correlation (R^2^=0.690) in patients with p.Phe508del homozygote genotype and sex.

The consanguinity rate was 92% in the homozygous p.Phe508del patients. The mean age at diagnosis was lower than the other genotypes (70.92±9.15 vs. 116.5±11.58 months) and it was further decreased in patients with complex genotype (5.625±1.84 months). The mean sweat sodium chloride concentration was higher in patients who had at least one mutation in p.Phe508del alleles in comparison with patients who carried other mutations except p.Phe508del (128.25±4.51 vs 99.2±4.25). There was no difference between pulmonary signs and symptoms of disease in patients carrying the p.Phe508del mutation compared to the patients who did not carry this mutation. Infection or colonization with bacteria such as *Escherichia coli* (*E. coli*) and *Enterococcus* was also more common in them.

### Genotype-phenotype correlation

Six patients were heterozygous for p. [Arg117His; 5T] and one of them was homozygous. The genotype of five patients (8%) was p. [Phe508del]; [Arg117His]; two of them had shown complex genotype, the genotype of one of them was p. [Phe508del]; [Arg117His] ; [Trp1282X] and the other one (1.6%) was p. [Phe508del]; [Phe508del]; [Arg117His]; [Arg117His]; [Gly542X]. The genotype of one patient (1.6%) was p. [Arg117His]; [Asn1303Lys]. All of them carried 5T variant. Five patients (8%), carried 7T variant, were heterozygous for p. Arg117His but second mutation was not detected in these groups of patients.

**Figure 2.**
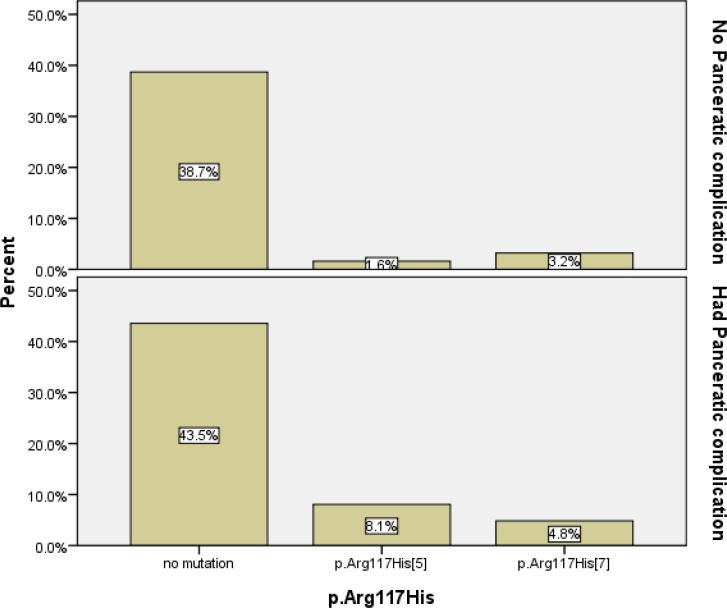
Relationship of p.Arg117His;[5T] mutation with insufficiency pancreatic in the CF patients registered at Pediatric Respiratory Disease Research Center of Iran.

We found a strong linear correlation (R^2^=0.864) between the age at diagnosis and p.Arg117His mutation carrying 7T variant. Although, patients with [p. Arg117His]; [5T] genotype showed 5 times more (8.1 versus 1.6%) pancreatic insufficiency (Figure 2), there was not any correlation between the mutation in this codon and severity of disease.

The third most frequent mutation in this CF patient cohort study was p.Trp1282X (Table 2). Among 6 patients who had this mutation, 5 patients (83.3%) were compound heterozygotes, carrying p.Phe508del as well. The patient who was homozygote for p.Trp1282X also carried p.Phe508del and c.489+1G>T mutations. The genotype of one patient was p. [Trp1282X]; [?]. The frequency of the p.Gly542X allele was 4% in this study. Among 4 patients who had this mutation, one patient was homozygote and the other patients were heterozygotes. The genotype of one of them was p. [Ile507del]; [Gly542X]. The other two patients had a complex genotype because one patient also carried p. Phe508del and p.Arg117His with 5T variant and the other one was p. [Gly551Asp]; [(c.1585–1G>A)].

Statistical analysis did not show any difference between different kinds of mutations and severity of disease, but patients with complex genotypes showed severe disease more than the other patients (85.7 vs. 56.6%), because the Shwachman-Kulczycki score was lower than 25 in this group of patients.

## DISCUSSION

There are few studies on molecular genetics of CF in Iran, but we have for the first time undertaken a descriptive study investigating the common *CFTR* mutations in Iran and their relationship to the clinical phenotype seen in Iranian CF patients. The goals of this study were to determine the frequencies of the common *CFTR* mutations in CF patients who were registered at the Pediatric Respiratory Disease Research Center of Iran and to evaluate correlations between genotype and phenotype in this cohort of patients. Since the patients were all recruited at the National Referral Centre for Iranian CF, our cohort included patients from all geographic regions of Iran.

Our results showed that the most frequent mutation in Iran was p.Phe508del. A (33.9%). The frequency of the p.Phe508del mutation in this cohort was higher than previous reports in Iran (4,5,7,17), but lower than those reported in European countries and higher than eastern neighboring countries such as Pakistan and India (18).

It seems that Iran acts as a bridge between these regions. We have also shown that the frequency of p.Phe508del mutation in the Baloch ethnic group was higher than the other Iranian groups such as the Lore and the Turk. Our study confirms the findings of a previous study which also reported a higher prevalence of p.Phe508del mutation in the Baloch ethnic group (19). It seems that our result confirmed the Dawson’s hypothesis. Based on the Dawson’s hypothesis, the original founder p.Phe508del mutation that gives rise to CF, occurred in those inhabiting the Iranian Plateau and eventually travelled from there to Europe in the first wave of emigrants (20,21). The Iranian Plateau was said to be the original Baluchi homeland and they had migrated to their present location which is known as one of the most isolated regions in the world until the 14th century AD. The Baluchi people have remained as an isolated group living in a large area with traditional arranged consanguineous marriages (22). Direct transfer of the p.Phe508del mutation had been likely to have occurred in later emigration into the Punjab of India, Oman and the present UAE. It is not surprising, therefore, that about 75% of the patients we have seen of Baluch descent are homozygous for the mutation p.Phe508del. Additionally, we couldn’t find any CF patients in adjacent provinces such as Kerman, Yazd and Hormozgan. Thus, in an area close to Baluchistan, CF appears to be a very rare disease, yet it is now found regularly in Baluchis.

Since our center is a national referral center for pediatric respiratory disease, we serve a wide population from all regions of Iran, and most of our patients are referred with severe respiratory signs and symptoms of the CF, such as bronchiectasis (12). We think this is the most likely reason for the higher rate of p.Phe508del mutation in this group of patients. The second probable reason is the higher consanguinity rate in this study in comparison with similar studies in Iran (74.2 vs 60%) (4).

Our study showed that there was a significant difference between p.Phe508del mutation and gender of the patients (p<0.032; CI: 95%) which was more common in females. There was a positive correlation between these variables. Given that *CFTR* is located on an autosomal chromosome there is no clear explanation for this finding. Others have investigated this finding and William’s study demonstrated that while there was a significant difference in the proportions of ‘normal’ and ‘p.Phe508del’ sperm, the difference between p.Phe508del/X and p.Phe508del/Y sperm is not significant. The sex ratio distortion for CF carriers is due to events which occur pre-fertilization (23). It seems that there is a natural selection for p.Phe508del/X sperms in comparison to p.Phe508del/Y sperm from healthy carriers. Therefore, we saw an increased rate of sex ratio in patients carrying p.Phe508del mutation. It is obvious that more developed studies are needed to prove this hypothesis.

Although, statistical analysis did not reveal any correlation between the p.Phe508del mutation and younger age at diagnosis or higher sweat chloride concentration, we saw considerable differences in these variables in comparison with the other genotypes. We believe that, there would be a significant correlation between these variables if the sample size were larger. For this reason, we suggest a multi-centered study to address this issue. Additionally, our result showed that there was a correlation between p. [Phe508del]; [Phe508del] genotype with severity of disease. Since mutation in p. Phe508del causes a class II *CFTR* mutation, the result is not surprising and is consistent with other studies that have shown that class II mutations cause severe disease (8,24).

Based on our study frequency of p.Arg117His, which is a class IV mutation in *CFTR* gene, is more common than in European populations but the rate of heterozygote allele p.[Phe508del]; [Arg117His] was similar to France (3,25). To our knowledge there is only one report on the p.Arg117His *CFTR* mutation in Iranian patients, but the frequency of p.Arg117His mutation in our study was much higher (9.7 vs 1.3%) (7). It may be due to the limitation of their studied population. Their study was limited to population with CF from the Iranian Azeri Turkish origin. On the other hand, linear regression analysis showed that there was a strong correlation between p. [Arg117His]; [7T] and age at diagnosis. It is likely that this type of mutation causes milder disease and is associated with Congenital Bilateral Absence of Vas Deferens (CBVAD) (26). So the age of presentation, disease onset and consequently the diagnostic age are higher in cases with p. Arg117His mutation. Most other studies have demonstrated a relationship between pancreatic insufficiency and the p. [Arg117His]; [5T] (27). Based on our observations the patients who were p. [Arg117His]; [5T] suffered five times more from pancreatic insufficiency (Figure 2).

The third most commonly detected mutation in the CF patients registered at the Pediatric Respiratory Research Disease of Iran was p.Trp1282X. Our result also indicated that the frequency of p. Trp1282X mutation in Iranian CF patients was similar to worldwide reports and had a high frequency like other Mediterranean regions (28). A previous study showed a high frequency of mutation in this codon in Ashkenazi Jews (29). In comparison with Abeliovich’s study and based on p.Phe508del and p.Trp1282X mutations, it seems that Iranian CF patients are more similar to Arab and Sephardic Jews race than Ashkenazi Jews (29). According to hypothesis of Dawson and Frossard (20) as well as Morral et al., study (30), it seems that shared ancestry is reasonable.

In European populations, the p.Gly542X, p.Asn1303Lys and p.Gly551Asp mutations account for 10–15% of *CFTR* as well as p.Gly542X and p.Asn1303Lys which are common in Mediterranean regions (28).

The fourth and fifth most *CFTR* frequent mutations in our study were p.Gly542X and p.Asn1303Lys, respectively. This result was consistent with previous studies conducted in Iran and reported by World Health Organization (WHO) (2,4, 17). Although the results indicated a weak linear relationship between the mutation at pG542X and dyspnea on one side and between pN1303K with lung infiltration and hyperinflation on the other side, these are preliminary findings from a small data set and a large multi centered study is required.

Mutation in p.Gly551Asp and (c.1585–1G>A) was detected simultaneously in two patients. Both of them suffered from a severe disease. Previous studies conducted in Iran neither did consider these mutations nor found any mutation in these codons (2,4–7,31).

None of the studied cases showed any mutation in p.Arg553X and p.Ar560Thr. The result was similar to previous studies conducted in Iran (2,4–7,31). According to the opinion of the authors, there is possible to find the specific mutations in these codons of CFTR gene in Iranian patients. So it shall not be omitted from screening program of CF.

Since about 35% of the CF patients did not carry the *CFTR* mutations we tested, it is possible to find rare and specific mutations in the Iranian population based on complete sequences of the gene. It seems that the whole genome sequencing can reveal some other and new specific mutation in Iranian patients. Unfortunately, we couldn’t do that because of limitation of budget. But the whole genome sequencing of *CFTR* gene is recommended to find new mutations in this group of patients.

## CONCLUSION

The study showed a wide heterogeneity of mutation in the *CFTR* gene within the CF patients registered at the Pediatric Respiratory Disease Research Center of Iran. Based on p.Phe508del and p.Trp1282X mutations, it seems that Iranian CF patients are more similar to Arab and Sephardic Jews race than Ashkenazi Jews. Consequently, it is essential to design a national screening program. On the other hand, in order to find the relationship between genotype and phenotype in some codons and any logical correlation between the genotype and phenotype of disease in CF patients, an extensive and inclusive study has to be designed.
